# Highly Enantioselective Production of (*R*)-Halohydrins with Whole Cells of *Rhodotorula rubra* KCh 82 Culture

**DOI:** 10.3390/ijms151222392

**Published:** 2014-12-04

**Authors:** Tomasz Janeczko, Monika Dymarska, Edyta Kostrzewa-Susłow

**Affiliations:** Department of Chemistry, Wrocław University of Environmental and Life Sciences, Norwida 25, Wrocław 50-375, Poland; E-Mails: monika.dymarska@gmail.com (M.D.); ekostrzew@gmail.com (E.K.-S.)

**Keywords:** enantiospecific reduction, β_2_-adrenoceptor-stimulating agent, (*R*)-halohydrins, *Rhodotorula rubra*

## Abstract

Biotransformation of ten α-haloacetophenones in the growing culture of the strain *Rhodotorula rubra* KCh 82 has been carried out. Nine of the substrates underwent an effective enantioselective reduction to the respective (*R*)-alcohols according to Prelog’s rule, with the exception of 2-chloro-1,2-diphenylethan-1-one that was not transformed by this strain. The expected reduction proceeded without dehalogenation, leading to the respective (*R*)-halohydrins in high yields. The use of this biocatalyst yielded (*R*)-2-bromo-1-phenyl-ethan-1-ol (enantiomeric excess (ee) = 97%) and its derivatives: 4'-Bromo- (ee = 99%); 4'-Chloro- (ee > 99%); 4'-Methoxy- (ee = 96%); 3'-Methoxy- (ee = 93%); 2'-Methoxy- (ee = 98%). There were also obtained and characterized 2,4'-dichloro-, 2,2',4'-trichloro- and 2-chloro-4'-fluoro-phenyetan-1-ol with >99% of enantiomeric excesses.

## 1. Introduction

Optically active chlorohydrins are versatile intermediate products in synthesis of biologically active compounds of high importance in the pharmaceutical industry and in agriculture [1]. The important pharmaceuticals of this kind include denopamine, isoproterenol, formoterol and salmeterol, which are β-adrenergic receptor agonists (all of them with *R* configuration at the carbon with an OH group). Both formoterol and salmeterol are used in treatment of chronic obstructive pulmonary disease, being highly selective β_2_-adrenergic receptor agonists. They are administered via inhalation [2,3,4]. Denopamine is a selective β_1_-adrenergic agonist and a useful drug for congestive heart failure [5,6]. Whereas, isoproterenol (known also as isoprenaline) is a non-selective drug, which stimulates both β_1_- and β_2_-adrenergic receptors. It is used mainly to prevent cardiac arrhythmia that occurs when the electrical impulses to the heart are not working properly [7,8].

The key step in synthesis of β_2_-adrenergic receptor agonists of desired stereochemistry is enantiospecific reduction of respective α-haloacetophenones. This may be achieved either by chemical methods [9,10,11], or by biotechnological ones. The effective enantioselectivity of the desired product was achieved either by using whole cells of biocatalysts [12,13,14,15,16] or with the help of isolated enzymes, such as dehydrogenases [1,17,18] and lipases [19]. Although in the majority of the reported studies, the halohydrins were obtained with high substrate conversions and high enantiomeric excesses, there is always a risk of side products due to reductive dehalogenation (leading to respective 1-phenylehtan-1,2-diols) and substitution (2-hydroxyacetophenone and 1-phenylehtan-1,2-diol) [20,21].

These undesired processes were observed with higher percent of conversion for α-bromoacetophenone and its derivatives than for the analogous chloroacetophenones [20]. However, in synthesis of many adrenergic receptor agonists and other relative compounds bromohydrins are more useful than chlorohydrins, because a bromine atom is a better leaving group when substituted by an amine or other nucleophiles and also bromohydrins can be easier converted into epoxides (important intermediate products in synthesis of many pharmaceuticals) [22,23].

For our study on effective reduction of selected acetophenone chloro- and bromo derivatives, we have chosen *Rhodotorula rubra* KCh 82 strain, because of its known and described earlier ability to reduce low-molecular-weight ketones [24,25,26] and relatively low reversibility of this process [25].

The strains of this species are described in literature as expansive ones, characterized by high growth potential (even on low-cost culture media) and high production of carotenoids (a typical feature of this species is red color of cells) [27]. They are also reported as biocatalysts that possess *S*-specific dehydrogenases towards aliphatic-aromatic ketones [28,29,30] and ketophosphonates [31,32]. This microorganism is also capable of enantioselective hydrolysis of epoxides to lactones with high enantimeric excesses [33].

## 2. Results and Discussion

We performed reduction of ten substrates (mostly halogen derivatives) in the culture of the tested strain of *Rhodotorula rubra* KCh 82. It was only 2-chloro-1,2-diphenylethan-1-one that did not undergo biotransformation in the culture of this strain. The other substrates underwent an effective enantioselective reduction to respective (*R*)-alcohols, according to Prelog’s rule (Table 1).

The strains of the species *R*. *rubra* have already been employed for the enantioselective reduction of α-bromoacetophenone (**1a**) [34]. The process led to (*R*)-2-bromo-1-phenyl-ethan-1-ol (**2a**) with 61% enantiomeric excess and 70% yield. When an anionic surfactant was used (sodium lauryl sulfate) and the transformation was run under argon, the ee increased to 95% and the yield to 90%. Under the same conditions bioreductions of six other α-bromoacetophenones (3'-chloro, methyl, and benzyloxy derivatives) were carried out [34].

**Table 1:** Biotransformations of α-haloderivatives of acetophenone in the culture of *R. rubra* strain.

Reaction	Product	Time (Days)	Conversion (%) ^a^	ee (%) ^a^	Config.
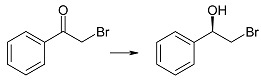	**2a**	1	>99	88	*R*
		3	>99	96	
		6	>99	97	
		9	>99	97	
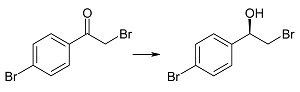	**2b**	1	>99	99	*R*
		3	>99	99	
		6	>99	99	
		9	>99	99	
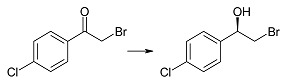	**2c**	1	85	>99	*R*
		3	95	96	
		6	>99	94	
		9	>99	93	
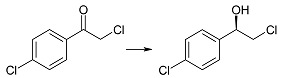	**2d**	1	97	96	*R*
		3	98	98	
		6	>99	99	
		9	>99	>99	
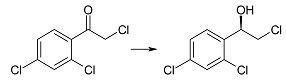	**2e**	1	>99	91	*R*
		3	>99	98	
		6	>99	>99	
		9	>99	>99	
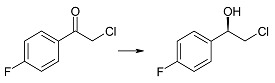	**2f**	1	>99	98	*R*
		3	>99	99	
		6	>99	>99	
		9	>99	>99	
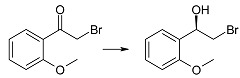	**2g**	1	>99	98	*R*
		3	>99	97	
		6	>99	97	
		9	>99	98	
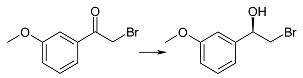	**2h**	1	>99	93	*R*
		3	>99	93	
		6	>99	93	
		9	>99	93	
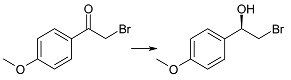	**2i**	1	>99	93	*R*
		3	>99	95	
		6	>99	96	
		9	>99	96	

^a^ Conversion and enantiomeric excesses were determined by GC analysis using chiral columns; and Config.: Configuration.

Using lyophilized cells of *Rhodotorula* sp. LSL suspended in water led to compound **2a** with ee > 99%, whereas adjusting the acidity of the solution to pH = 4.3 gave a product with >99% conversion [12]. The authors of this paper also described enantioselective reduction of five other α-halogen derivatives of acetophenone (including α-chloroacetophenone and its 4'-chloro and nitro derivatives, and also 3'-nitro and 4'-methoxy derivatives of α-bromoacetophenone). Therefore, taking into account all this information, we conclude that our results reported here make a good contribution to the widespread research on effective methods of receiving (*R*)-2-bromo-1-phenyl-ethan-1-ols. Additionally, they broaden the substrate spectrum of *R*. *rubra* strains and biocatalysis in general.

(*R*)-(−)-2-Bromo-1-(4'-bromophenyl)-ethan-1-ol (**2b**) was obtained in the culture of *Rhodotorula rubra* KCh 82 with >99% of substrate conversion and 99% of enantiomeric excess. For this substrate no changes in the conversion and enantiomeric excess were noticed during the biotransformation process (Table 1). It is known from literature that compound **2b** can be received with 51% yield and 94% ee by enantioselective esterification of racemic compound **2b** using lipase from *Pseudomonas fluorescens* [35]. There were reported also chemical methods of enantioselective reduction of 2-bromo-4'-bromoacetophenone (**1b**) to (*S*)-(+)-**2b** (89% yield and ee = 96%) using boron compounds modified with chiral phosphoric acid derivatives [36] or using BH_3_ modified with *S*-proline derivatives (99% yield and ee = 46%) [37]. It is also known that compound **2b** was obtained by reduction of the respective ketone in the cultures of five *Yarrowia lipolytica* strains [38]. In the culture of the strain *Y*. *lipolytica* ATCC 32-338A after three days of incubation with the substrate (*R*)-alcohol **2b** was received with both conversion and enantiomeric excess of over 99% (after one day the conversion was below 65%).

**Figure 1:**
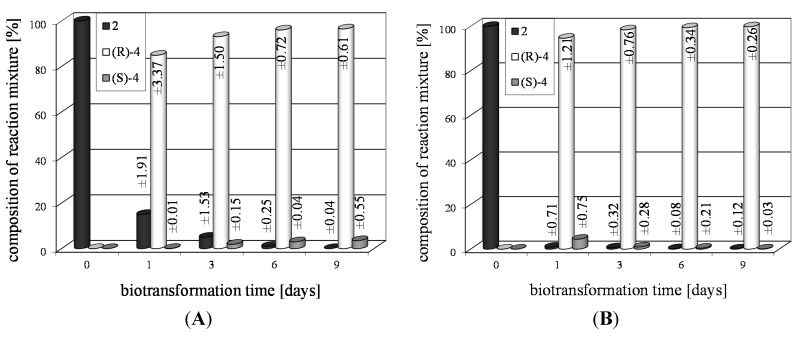
Time dependence of transformation of: (**A**) 2-bromo-4'-chloroacetophenone (**1c**) and (**B**) 2,2',4'-trichloroacetophenone (**1e**) in the culture of *Rhodotorula rubra* KCh 82.

Both (*S*)-(+)- as well as (*R*)-(−)-2-bromo-1-(4'-chlorophenyl)-ethan-1-ol (**2c**) can be obtained by chemical methods [36,39]. In literature there are twenty microbial strains described that can reduce 2-bromo-4'-chloroacetophenone (**1c**) to compound **2c** [38,40]. Among them, the highest substrate conversion (89%) was noted in the culture of *Candida magnoliae* IFO11, however, the enantiomeric excess of the alcohol obtained was only 68%. The enantiomeric excesses of over 95% were obtained in the cultures of seven biocatalysts and among them the highest substrate conversion (67%) was observed for *Rhodotorula glutinis* var. *dairenensis* IFO415 [40]. In the culture of the strain tested by us, *Rhodotorula rubra* KCh 82, we observed a drop in enantiomeric excess of product (**2c**) during biotransformation process. After one day of the reaction this alcohol was formed with ee = 99% (conversion 85%), however, during biotransformation time the conversion increased, but the enantiomeric excess decreased (Figure 1A). After six days of incubation the conversion reached >99%, but the ee dropped to 94%. Such a course of the biotransformation is the result of the enantioselective reduction of the ketone to the *R*-alcohol **2c**, and then even more enantioselective oxidation of the *R*-alcohol.

The opposite change in enantioselectivity of the reduction was observed during incubation of 2-chloro-4'-chloroacetophenone (**1d**) in the culture of the tested strain. In this case after one day of biotransformation (*R*)-(−)-2-chloro-1-(4'-chlorophenyl)-ethan-1-ol (**2d**) was observed with ee = 96% and conversion = 97%. Both the conversion and enantiomeric excess increased with biotransformation time (>99% after nine days, Table 1). It is also possible to obtain compound (*R*)-**2d** by enzymatic methods, using commercially available keto reductases, but only with the help of coenzyme NADPH [41].

Whereas, (*S*)-**2d** (ee = 96.6%) was reported to be obtained by microbiological methods from substrate **1d**, using the strain *Geotrichum* sp. 38, though in low yield (42%) [42]. The *R*-isomer of alcohol **2d** can be obtained also in the culture of *Yarrowia lipolytica* ATCC 32-338A, however, with rather low conversion (27%) and ee = 67%, after nine days of biotransformation [38]. The most effective was the process catalyzed by lyophilized cells of *Rhodotorula* sp. LSL suspended in water solution (pH = 4.3), conducted under argon, which gave >99% of both conversion and ee [12].

2-Chloro-1-(2',4'-dichlorophenyl)-ethan-1-ol (**2e**) was obtained in 97%–99% yield and with 90%–92% of ee by chemical synthesis, using triethylamine and chiral ruthenium complexes [43]. The authors of that work did not establish the absolute configuration of the product. There are also reports on reduction of **1e** to **2e** in the cultures of the strains of *Yarrowia lipolytica*. The most effective was the strain *Y*. *lipolytica* A50, which gave product **2e** with >99% of conversion and ee = 48% after nine days of the reaction [38]. In the culture of the strain *Rhodotorula rubra* KCh 82 described here the (*R*)-alcohol **2e** was received with >99% of conversion after one day of the transformation (ee = 91%). Extending the reaction time to six days gave pure (*R*)-(−)-alcohol (**2e**) as a single reaction product (Figure 1B).

Similar enantioselectivity was observed also for biotransformation of 2-chloro-4'-fluoroacetophenone (**1f**) in the culture of the tested *R*. *rubra* KCh 82 strain. The respective (*R*)-alcohol **2f** with ee > 99% was obtained after six days of biotransformation (Table 2). In literature it has been reported that (*R*)-(−)-2-chloro-1-(4'-fluorophenyl)-ethan-1-ol (**2f**) (ee = 99%) was received by enzymatic reduction of compound **1f** using alcohol dehydrogenase (YMR226c) from *Saccharomyces cerevisiae* yeasts in the presence of coenzyme NADPH [44]. Product **2f** was also obtained in biotransformation of **1f** in the culture of *Y*. *lipolytica* ATCC 32-338A, however, with low conversion (12%) and ee = 48% [38]. *S-* and *R*-enantiomer of compound **2f** were also received by kinetic separation with the use of lipase (Amano PS-C). This enzyme afforded (*R*)-**2f** with 53% yield and ee = 85%, along with the acetate of alcohol (*S*)-**2f** in 47% yield and ee = 95% [45].

Three methoxy derivatives of α-bromoacetophenone (**1g**–**i**) were effectively reduced by *Rhodotorula rubra* KCh 82 (after one day of transformation the substrates were fully consumed) and we observed that the enantiomeric excesses of the respective alcohols underwent small changes during biotransformation time (Table 1). To the best of our knowledge there have been no reports on microbial reduction of 2'-methoxy and 3'-methoxy α-bromoacetophenone (**1g** and **1h**), so far, (*S*)-(+)-2-bromo-1-(2'-methoxyphenylo)-ethan-1-ol (**2g**) can be synthesized with 99% of conversion and ee = 86% by reduction of 2-bromo-2'-methoxyacetophenone (**1g**) with lithium borohydride modified with chiral boron compound [46]. Racemic 2-bromo-1-(3'-methoxyphenyl)-ethan-1-ol (**2h**) was obtained by reduction of 2-bromo-3'-methoxyacetophenone (**1h**) with water/dioxane NaBH_4_ solution [47].

After one day of incubation of 2-bromo-4'-methoxyacetophenone (**1i**) in the culture of the tested *R*. *rubra* KCh 82 strain we observed the desired *R*-alcohol **2i** with the enantiomeric excess of 93%. Due to the activity of dehydrogenases of this biocatalyst the ee of the product increased to 96% during biotransformation time. In literature it is known a synthetic method of receiving 2-bromo-1-(4'-methoxyphenyl)-ethan-1-ol (**2i**) by enantioselective reduction of **1i** with chiral ruthenium compounds [48]. The product was obtained with 82% of conversion and 98% of enantiomeric excess (no data about the configuration, though). In this method the products of dehalogenation: compound **1i** and **2i** were also observed, as 18% altogether of the reaction mixture content. It is possible to obtain (*R*)-alcohol **2i** with the enantiomeric excess of 77%–87% by enantioselective esterification of racemic **2i** with the help of lipases from *Pseudomonas* sp. (PS-C) and *Pseudomonas fluorescens* [45,49]. The same alcohol (*R*)-**2i** was also obtained with >99% of both conversion and the ee using lyophilized cells of *Rhodotorula* sp. LSL suspended in aqueous solution of pH = 4.3 [12].

## 3. Experimental Section

### 3.1. Materials

All substrates were purchased from Sigma-Aldrich (St. Louis, MO, USA). The *Rhodotorula rubra* KCh 82 strain was obtained from the Department of Chemistry of Wrocław University of Environmental and Life Sciences (Wrocław, Poland). The strain was cultivated on a Sabouraud agar consisting of aminobac (5 g), peptone K (5 g), glucose (40 g) and agar (15 g) dissolved in 1 L of distilled water, at 25 °C and pH 6.5 and stored in a refrigerator at 4 °C.

### 3.2. Analytical Methods

The course of biotransformation was controlled by means of Thin Layer Chromatography (TLC). Analytical TLC was carried out on silica gel G 60 F_254_ plates (Merck, Darmstadt, Germany). Chromatograms were developed using hexane/acetone mixture (3:1 *v*/*v*) as the eluent. Compounds were detected by spraying the plates with 1% Ce(SO_4_)_2_ and 2% H_3_[P(Mo_3_O_10_)_4_] in 10% H_2_SO_4_. The products were separated by column chromatography using silica gel (SiO_2_, Kieselgel 60, 230–400 mesh, 40–63 μm, Merck) and hexane/acetone mixture (3:1, *v*/*v*) as the developing system. Composition of biotransformation mixtures was established by gas chromatography (GC) on Agilent Technologies 7890 A GC instrument (Santa Clara, CA, USA), fitted with a flame ionization detector (FID) and a chiral column Chirasil-Dex CB (Agilent) 25 m × 0.25 mm × 0.25 μm film thickness. Temperature of injector: 200 °C; temperature of detector: 250 °C was the same for all compounds. To determinate the composition and enantiomeric excesses of product mixtures the following temperature programs were used (Table 2).

Reference samples of the racemic alcohols were prepared by reducing the ketones with sodium borohydride in methanol. NMR spectra were recorded on a DRX 600 MHz Bruker spectrometer (Bruker, Billerica, MA, USA) and measured in CDCl_3_. Optical rotations were measured with an Autopol IV automatic polarimeter (Rudolph, Hackettstown, NJ, USA). Absolute configurations of the products were determined by comparison of their optical rotation values with literature data.

**Table 2:** Temperature programs (°C) used for gas chromatography.

Compound Number	Starting T (°C) 1 min	Gradient (°C·min^−1^)	T (°C) 0 min	Gradient (°C·min^−1^)	Final T (°C) 5 min	*R*_t_ of *S-*Isomer (min)	*R*_t_ of *R-*Isomer (min)
**2a**	102	1.5	126	20	200	19.2	19.5
**2b**	150	2	177	20	200	11.4	11.8
**2c**	147	2	175	20	200	9.3	9.8
**2d**	147	2	165	20	200	8.2	8.7
**2e**	150	3	185	20	200	7.6	8.2
**2f**	140	2	153	20	200	4.1	4.5
**2g**	130	3	155	20	200	10.8	11.1
**2h**	120	0.1	124	20	200	45.3	45.8
**2i**	120	3	155	20	200	14.4	14.7

T: Temperature.

### 3.3. Screening Procedure

Erlenmeyer flasks (300 mL), each containing 100 mL of the medium consisting of 3 g glucose and 1 g aminobac dissolved in water, were inoculated with a suspension of microorganisms and then incubated for 3–7 days at 25 °C on a rotary shaker (190 rpm). After full growth of the culture 20 mg of a substrate dissolved in 1 mL of acetone was added. After 1, 3, 6, 9 days of incubation under the above conditions, portions of 10 mL of the transformation mixture were taken out and extracted with CHCl_3_ (3 × 10 mL). The extracts were dried over MgSO_4_, concentrated *in vacuo* and analyzed by GC. All the experiments were repeated three times.

### 3.4. Preparative Biotransformation

The same transformations were performed on the preparative scale in 2000 mL flasks, each containing 500 mL of the cultivation medium. The cultures were incubated under the same conditions and then 200 mg of substrates dissolved in 10 mL of acetone were added to the grown cultures. After incubation the mixtures were extracted with CHCl_3_ (3 × 300 mL), dried (MgSO_4_) and concentrated *in vacuo*. The transformation products were separated by column chromatography and analyzed (TLC, GC, and also confirmed by ^1^H NMR).

### 3.5. Spectral Data of Isolated Metabolites

(*R*)-(−)-2-Bromo-1-phenylethan-1-ol (**2a**): A three-day transformation of substrate **1a** (100 mg) in the culture *R. rubra* KCh 82 yielded 91 mg of compound **2a**: ${\left[\alpha \right]}_{D}^{25}$ = −30.1° (*c* = 1.32 CHCl_3_) (97% ee), ([49], ${\left[\alpha \right]}_{D}^{25}$ = −33°, 93% ee); ^1^H NMR (CDCl_3_) δ: 2.68 (s, 1H, –O*H*), 3.59 (dd, 1H, *J* = 10.5, 9.0 Hz, one of –C*H*_2_–), 3.68 (dd, 1H, *J =* 10.5, 3.3 Hz, one of –C*H*_2_–), 4.96 (dd, 1H, *J* = 9.0, 3.3 Hz, –C*H*OH–), and 7.35–7.45 (m, 5H, H–Ar).

(*R*)-(−)-2-Bromo-1-(4'-bromophenyl)-ethan-1-ol (**2b**): A one-day transformation of substrate **1b** (100 mg) in the culture *R. rubra* KCh 82 yielded 93 mg of compound **2b**:

$${\left[\alpha \right]}_{D}^{25}$$

= −41.6° (*c* = 14.1 CHCl_3_) (99% ee), ([35], ${\left[\alpha \right]}_{D}^{25}$ = +56.6°, 95% ee); ^1^H NMR (CDCl_3_) δ: 2.72 (s, 1H, –O*H*), 3.52 (dd, 1H, *J* = 10.5, 8.8 Hz, one of –C*H*_2_–), 3.62 (dd, 1H, *J* = 10.5, 3.4 Hz, one of –C*H*_2_–), 4.91 (dd, 1H, *J* = 8.8, 3.4 Hz, –C*H*OH–), 7.28–7.38 (m, 2H, H-3' and H-5'), and 7.53–7.59 (m, 2H, H-2' and H-6').

(*R*)-(−)-2-Bromo-1-(4'-chlorophenyl)-ethan-1-ol (**2c**): A three-day transformation of substrate **1c** (100 mg) in the culture *R. rubra* KCh 82 yielded 79 mg of compound **2c**: ${\left[\alpha \right]}_{D}^{25}$ = −36.5° (*c* = 3.94 CHCl_3_) (98% ee), ([50], ${\left[\alpha \right]}_{D}^{25}$ = +39.0°, 89% ee); ^1^H NMR (CDCl_3_) δ 2.80 (s, 1H, –O*H*), 3.52 (dd, 1H, *J* = 10.5, 8.8 Hz, one of –C*H*_2_–), 3.61 (dd, 1H, *J* = 10.5, 3.4 Hz, one of –C*H*_2_–), 4.92 (dd, 1H, *J* = 8.8, 3.4 Hz, –C*H*OH–), and 7.36–7.48 (m, 4H, H–Ar).

(*R*)-(−)-2-Chloro-1-(4'-chlorophenyl)-ethan-1-ol (**2d**): A three-day transformation of substrate **1d** (100 mg) in the culture *R. rubra* KCh 82 yielded 93 mg of compound **2d**: ${\left[\alpha \right]}_{D}^{25}$ = −47.1° (*c* = 1.47 CHCl_3_) (99% ee), ^1^H NMR (CDCl_3_) δ 2.71 (s, 1H, –O*H*), 3.63 (dd, 1H, *J* = 11.3, 8.6 Hz, one of –C*H*_2_–), 3.73 (dd, 1H, *J =* 11.3, 3.3 Hz, one of –C*H*_2_–), 4.90 (dd, 1H, *J* = 8.6, 3.3 Hz –C*H*OH–), and 7.36–7.59 (m, 4H, H–Ar).

(*R*)-(−)-2-Chloro-1-(2',4'-dichlorophenyl)-ethan-1-ol (**2e**): A one-day transformation of substrate **1e** (100 mg) in the culture *R. rubra* KCh 82 yielded 93 mg of compound **2e**: ${\left[\alpha \right]}_{D}^{25}$ = −31.9° (*c* = 2.55 CHCl_3_) (99% ee), ([1], ${\left[\alpha \right]}_{D}^{25}$ = −52.8°, 99% ee); ^1^H NMR (CDCl_3_) δ: 2,84 (s, 1H, –O*H*), 3.55 (dd, 1H, *J* = 11.3, 8.5 Hz, one of –C*H*_2_–), 3.90 (dd, 1H, *J =* 11.3, 2.8 Hz, one of –C*H*_2_–), 5.29 (dd, 1H, *J* = 8.5, 2.8 Hz, –C*H*OH–), 7.34 (dd, 1H, *J* = 8.4, 2.1 Hz, H-5'), 7.40 (d, H1, *J* = 2.1 Hz, H-6'), and 7.60 (d, 1H, *J* = 8.4 Hz, H-3').

(*R*)-(−)-2-Chloro-1-(4'-fluorophenyl)-ethan-1-ol (**2f**): A one-day transformation of substrate **1f** (100 mg) in the culture *R. rubra* KCh 82 yielded 88 mg of compound **2f**: ${\left[\alpha \right]}_{D}^{25}$ = −38.6° (*c* = 1.10 CHCl_3_), (98% ee) ([51], ${\left[\alpha \right]}_{D}^{25}$ = +51.1°, 99% ee), ^1^H NMR (CDCl_3_) δ 2.62 (s, 1H, –O*H*), 3.65 (dd, 1H, *J* = 11.3, 8.8 Hz, one of –C*H*_2_–), 3.74 (dd, 1H, *J* = 11.3, 3.5 one of Hz, –C*H*_2_–) 4.74 (dd, 1H, *J* = 8.8, 3.5 Hz, –C*H*OH–), 7.09 (m, 2H *W*_h_ = 23.3 Hz, H-3' and H-5'), and 7.40 (m, 2H, *W*_h_ = 19.6 Hz, H-2' and H-6').

(*R*)-(−)-2-Bromo-1-(2'-methoxyphenyl)-ethan-1-ol (**2g**): A one-day transformation of substrate **1g** (100 mg) in the culture *R. rubra* KCh 82 yielded 88 mg of compound **2g**: = ${\left[\alpha \right]}_{D}^{25}$ 15.3°, (*c* = 1.26 CHCl_3_), (98% ee), ^1^H NMR (CDCl_3_) δ: 2.93 (s, 1H, –O*H*), 3.56 (dd, 1H, *J* = 10.2, 8.6 Hz, one of –C*H*_2_–), 3.79 (dd, 1H, *J* = 10.2, 3.6 Hz, one of –C*H*_2_–), 3.89 (s, 3H, –OC*H*_3_), 5.18 (dd, 1H, *J* = 8.6, 3.6 Hz –C*H*OH–), 6.92 (d, 1H, *J* = 8.2 Hz, H-3'), 7.02 (td, 1H, *J* = 7.5, 0.7 Hz, H-5'), 7.33 (td, 1H, *J* = 8.2, 1.6 Hz, H-4'), and 7.47 (dd, 1H, *J* = 7.5, 1.6 Hz, H-6').

(*R*)-(−)-2-Bromo-1-(3'-methoxyphenyl)-ethan-1-ol (**2h**): A one-day transformation of substrate **1h** (100 mg) in the culture *R. rubra* KCh 82 yielded 73 mg of compound **2h**: ${\left[\alpha \right]}_{D}^{25}$ = −12.8° (*c* = 1.36 CHCl_3_) (94% ee); ^1^H NMR (CDCl_3_) δ: 2.52 (s, 1H, –O*H*), 3.56 (dd, 1H, *J* = 10.5, 9.0 Hz, one of –C*H*_2_–), 3.66 (dd, 1H, *J* = 10.5, 3.3 Hz, one of –C*H*_2_–), 3.84 (s, 3H, –OC*H*_3_), 4.93 (dd, 1H, *J* = 9.0, 3.3 Hz, –C*H*OH–), 6.89 (ddd, 1H, *J* = 8.3, 2.4, 1.1 Hz, H-4'), 6.98–7.09 (m, 2H, H-2' and H-6'), and 7.32 (t, 1H,* J* = 8.3 Hz, H-5').

(*R*)-(−)-2-Bromo-1-(4'-methoxyphenyl)-ethan-1-ol (**2i**): A one-day transformation of substrate **1i** (100 mg) in the culture *R. rubra* KCh 82 yielded 76 mg of compound **2i**: ${\left[\alpha \right]}_{D}^{25}$ = −20.3° (*c* = 1.96 CHCl_3_), (96% ee); ([52], ${\left[\alpha \right]}_{D}^{25}$ = +19.6°, 96% ee for enatiomer *S*); ^1^H NMR (CDCl_3_) δ: 2.61 (s, 1H, –O*H*), 3.56 (dd, 1H, *J* = 10.4, 9.0 Hz, one of –C*H*_2_–), 3.63 (dd, 1H, *J* = 10.4, 3.4 Hz, one of –C*H*_2_–), 3.84 (s, 3H, –OC*H*_3_), 4.91 (dd, 1H, *J* = 9.0, 3.4 Hz –C*H*OH–), 6.92–6.95 (m, 2H, H-3' and H-5'), and 7.33–7.36 (m, 2H, Hz, H-2' and H-6').

## 4. Conclusions

The presented results revealed that the strain *Rhodotorula rubra* KCh 82 used for the biotransformations of ten 2-halogen derivatives of acetophenone showed high *R*-selectivity of the reduction of the substrates. With the help of this biocatalyst, we obtained nine (*R*)‑halohydrins with high both yields and enantiomeric excesses, which were fully characterized spectroscopically and are potential synthons for synthesis of β-adrenergic receptor agonists. It should be underlined that (*R*)-halohydrins were single products of these reactions. No side products were observed.
